# Creating customized oral stents for head and neck radiotherapy using 3D scanning and printing

**DOI:** 10.1186/s13014-019-1357-2

**Published:** 2019-08-19

**Authors:** Mohamed Zaid, Nimit Bajaj, Hannah Burrows, Ryan Mathew, Annie Dai, Christopher T. Wilke, Stephen Palasi, Ryan Hergenrother, Caroline Chung, Clifton D. Fuller, Jack Phan, G. Brandon Gunn, William H. Morrison, Adam S. Garden, Steven J. Frank, David I. Rosenthal, Michael Andersen, Adegbenga Otun, Mark S. Chambers, Eugene J. Koay

**Affiliations:** 1 Department of Radiation Oncology, Division of Radiation Oncology, The University of Texas MD Anderson Cancer Center, 1515 Holcombe Blvd, Unit 0097, Houston, TX 77030 USA; 2 Department of Radiation Oncology, University of Minnesota Medical School, 516 Delaware St SE, Minneapolis, MN 55455 USA; 3 Department of Head and Neck Surgery, Division of Surgery, The University of Texas MD Anderson Cancer Center, 1515 Holcombe Blvd, Houston, TX 77030 USA

**Keywords:** Radiation therapy, 3D printing, 3D scanning, Oral stents, Head and neck cancer

## Abstract

**Background:**

To evaluate and establish a digital workflow for the custom designing and 3D printing of mouth opening tongue-depressing (MOTD) stents for patients receiving radiotherapy for head and neck cancer.

**Methods:**

We retrospectively identified 3 patients who received radiation therapy (RT) for primary head and neck cancers with MOTD stents. We compared two methods for obtaining the digital impressions of patients’ teeth. The first method involved segmentation from computed tomography (CT) scans, as previously established by our group, and the second method used 3D scanning of the patients’ articulated stone models that were made during the conventional stent fabrication process. Three independent observers repeated the process to obtain digital impressions which provided data to design customized MOTD stents. For each method, we evaluated the time efficiency, dice similarity coefficient (DSC) for reproducibility, and the 3D printed stents’ accuracy. For the 3D scanning method, we evaluated the registration process using manual and automatic approaches.

**Results:**

For all patients, the 3D scanning method demonstrated a significant advantage over the CT scanning method in terms of time efficiency with over 60% reduction in time consumed (*p* < 0.0001) and reproducibility with significantly higher DSC (*p* < 0.001). The printed stents were tested over the articulated dental stone models, and the trueness of fit and accuracy of dental anatomy was found to be significantly better for MOTD stents made using the 3D scanning method. The automated registration showed higher accuracy with errors < 0.001 mm compared to manual registration.

**Conclusions:**

We developed an efficient workflow for custom designing and 3D-printing MOTD radiation stents. This workflow represents a considerable improvement over the CT-derived segmentation method. The application of this rapid and efficient digital workflow into radiation oncology practices can expand the use of these toxicity sparing devices to practices that do not currently have the support to make them.

**Electronic supplementary material:**

The online version of this article (10.1186/s13014-019-1357-2) contains supplementary material, which is available to authorized users.

## Background

Worldwide, head and neck cancer (HNC) accounts for more than 830,000 new cases per year with a mortality rate exceeding 430,000 [1]. A multidisciplinary approach is required for an optimal therapeutic strategy, and radiation therapy (RT) has demonstrated significant benefits in local tumor control and patient survival [2]. However, RT is challenged by its inherent toxicity, notably radiation induced oral mucositis (RIOM) [3]. In a systematic review, 80% of 6181 HNC patients who received RT developed RIOM, half of which were of severe forms (grade III and IV) [4]. RIOM significantly detracts from patients’ quality of life, and may result in unplanned treatment breaks or a change in the therapeutic regimens [5, 6]. Therefore, therapeutic modalities and devices have been developed to tackle RIOM, and one of the most utilized devices is the oral radiation stent. They effectively displace and immobilize healthy tissue away from the radiation path, thereby improving the therapeutic index [7–12]. However, the workflow for stent fabrication and the degree of customization varies. Previously, we demonstrated the feasibility of utilizing computer-aided-designing (CAD) and 3D-printing to create oral stents using routine diagnostic CT imaging studies [13]. However, this method is limited by availability of high quality scans, absence of dental artifacts, subjectivity in delineation of the dental anatomy, and bite registration inaccuracy, as the mandible is rotated around an anatomical average rather than a patient specific axis. These limitations lead to inaccurate and ill-fitting oral stents. Here, we introduce a novel workflow for the design and fabrication of customized oral stents using 3D-scanning technology. We investigated the utility and limitations in acquiring accurate and reproducible teeth impressions, and subsequent performance of 3D printed customized MOTD stents.

## Methods

### Patient population

We identified 3 patients (age 35–66, 2 males and 1 female) diagnosed with primary HNC (2 with oropharyngeal cancer, 1 with paranasal sinuses cancer) who received definitive RT (Table.1). All patients underwent head and neck CT scans for diagnostic purposes. Patients were referred to the Oral Oncology Department at The University of Texas MD Anderson Cancer Center (MDACC) for fabrication of MOTD stents, which are commonly used for base of tongue tumors at our institution [8] . This study was approved by the Institutional Review Board at MDACC under protocol 2017–0269.

**Table 1:** Demographics of the patients population

Age	Sex	Race	Tumor location	Tumor type	Stage
50	Male	Hispanic	Base of the tongue	Squamous cell carcinoma	T2 N2 M0
66	Female	White	Base of the tongue	Squamous cell carcinoma	T1 N1 M0
35	Male	Black	Nasal sinus	Undifferentiated carcinoma	T2 N0 M0

### CT imaging derived dental impressions

**Fig. 1:**
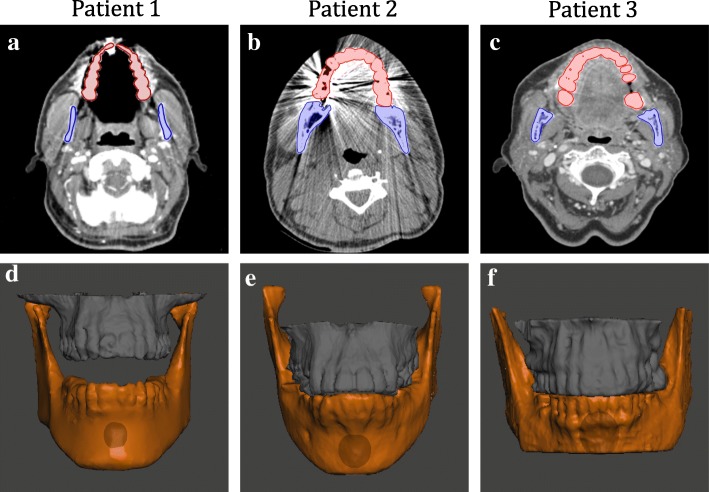
Manual segmentation of maxilla and mandible on pre-treatment CT scans of 3 patients (**a**, **b**, **c**) and 3D reconstruction of the segmented bone and teeth (**d**, **e**, **f**)

Diagnostic CT images of patients were acquired using a multidetector helical CT scanner (GE-Medical Systems, Milwaukee, WI). The slice thickness was 1-mm for two patients and 2.5-mm for the third. Three trained researchers independently segmented the maxillary and the mandibular anatomy (bones and teeth) using Velocity AI software (Varian Medical Systems, Palo Alto, CA) (Fig. 1a–c). To prevent bias, the observers were blinded to the results, measurements of the other observers, and the dental stone models which served as the ground truth**.** Time spent performing segmentation was recorded. To evaluate the segmentation reproducibility between the 3 observers, we calculated the dice similarity coefficient (DSC) [14] using 3D slicer software (http://www.slicer.org). Finally, we randomly selected one segmentation per patient to export as a stereolithography (STL) file, which was imported into a 3D-modeling software, Meshmixer (Autodesk Inc., San Rafael, CA) (Fig. 1d–f).

### 3D-scanning of the stone model and bite registration

**Fig. 2:**
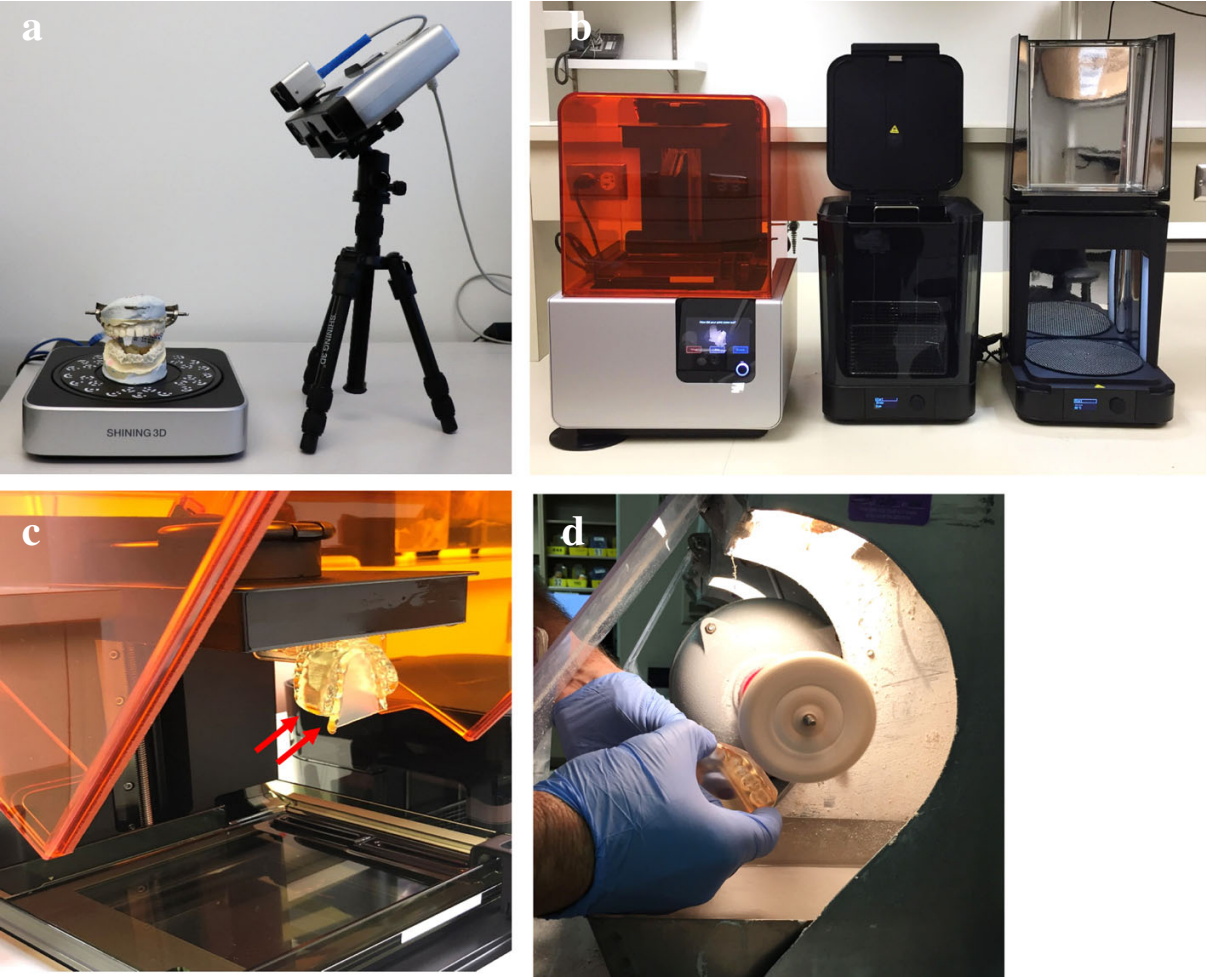
3D-printing process: **a** Dental stone model placed on the rotating platform of EinScan-Pro desktop scanner **b** FormLabs Form 2 printer, Form Wash and Form Cure unit assembly **c** A 3D-printed stent attached (red arrows) to the build platform of Form 2 printer **d** Stent polishing using ground pumice stone and rag wheel apparatus

The articulated dental stone models of the patients were obtained from the dental laboratory. These models were made by the oral-maxillofacial surgeons to manually fabricate the traditional radiation stent. Models were made through irreversible hydrocolloid dental impressions of the patients, which were poured in gypsum stone and articulated in the appropriate maxillo-mandibular jaw positioning (inter-incisal opening of 20 mm) using patient-specific jaw relation records made with Aluwax (Aluwax Dental Products Company, Allendale, MI.). We used a desktop white light 3D-scanner, EinScan-Pro (Shining 3D, Hangzhou, China), to individually scan the maxillary and mandibular models which provided the topographic occlusal anatomy, and the combined articulated stone models which provided the bite record (Fig. 2a). To validate the reproducibility of 3D-scanner, we repeated the scanning procedure three times per model, and calculated the DSC for the resulting volumes. To register the individual maxillary and mandibular meshes to the articulated mesh, we developed two different methods. 1) Manual registration, where we used Meshmixer software to maneuver the structures until a visually acceptable fit was achieved. 2) Automatic registration, using MeshLab 1.2.1 software (ISTI-CNR, Italy) which utilizes an Iterative Closest Point (ICP) algorithm [15]. For this we identified a minimum of 4 fiduciary points on individual and articulated meshes separately with the ‘point-based gluing’ method that aligns each reference mesh to the target mesh. These aligned meshes were then processed using preset ICP algorithm parameters for a highly accurate fit (errors < 0.001 mm). We repeated each method three times to compare time efficiency and accuracy.

#### Stent designing and fabrication

**Fig. 3:**
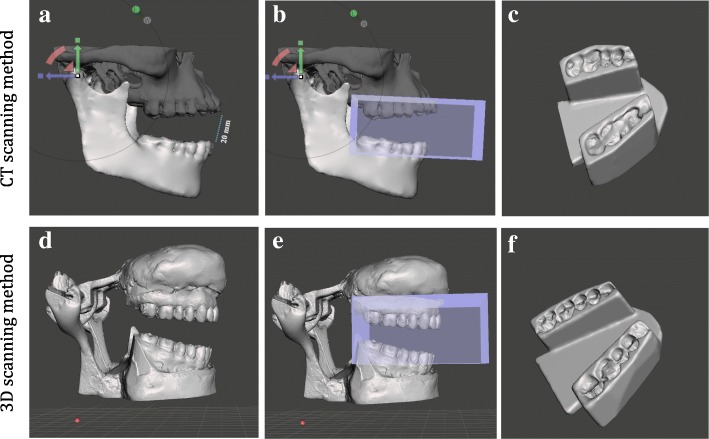
Workflow for digital stent design using CT-derived STL files (**a–c**), and the 3D-scanning method (**d–f**). **a** & **d** Mandibular rotation at Temporomandibular joint (TMJ) to achieve 20 mm inter-incisal opening; **b** & **e** Acquiring negative impression of the teeth; **c** & **f** Final completed stent

To create a CAD model of the MOTD stent, we followed the same steps we described previously [13]. The digital impression of the patient’s dentition was acquired from the registered maxillary and mandibular arches, once using the CT segmented volumes and the 3D scanned volumes each. This was followed by placing strategic custom cuts to remove excess material to have the desired shape of an MOTD stent (Fig. 3). Then, the designed stent files were imported into the PreForm software 2.18.0 (FormLabs Inc., Somerville, MA) and specifically oriented to minimize the interference of supporting struts on the occlusal and lingual surfaces. We used the Form 2 printer (FormLabs Inc., Somerville, MA) and Dental SG resin to print the stents (Fig. 2b, c). The 3D-printing process took approximately 4.5 h/stent and the layer thickness was 100 μm. Stents were then washed with 90% isopropyl alcohol (IPA) for 10 min to remove uncured resin; then completely ultraviolet (UV) cured using Form Cure machine (FormLabs Inc., Somerville, MA) for 30 min at 60 degrees Celsius to achieve optimal mechanical properties of the resin. Final finishing and polishing was accomplished using a dental laboratory lathe machine and pumice powder with rag wheel apparatus (Fig. 2d). Additional file 1: Figure S1 flowchart shows the comparison of both these methods.

## Results

### Time and reproducibility (CT vs. 3D-scanning)

Results showed a statistically significant difference (t-test, *p* < 0.0001) in the time consumed for acquiring the digital dental impressions between the two methods. The average time per observer was 40 min (range = 30–48, SD = 6.03) for segmentation of CT scans, and a constant 15 min for the 3D-scanning of a stone model. For reproducibility, the 3D-scanning method showed a significantly higher DSC (t-test, *p* < 0.0001) compared to the CT segmentation method (Additional file 2: Table S1 and Additional file 1: Figure S2).

### Stone model registration

**Fig. 4:**
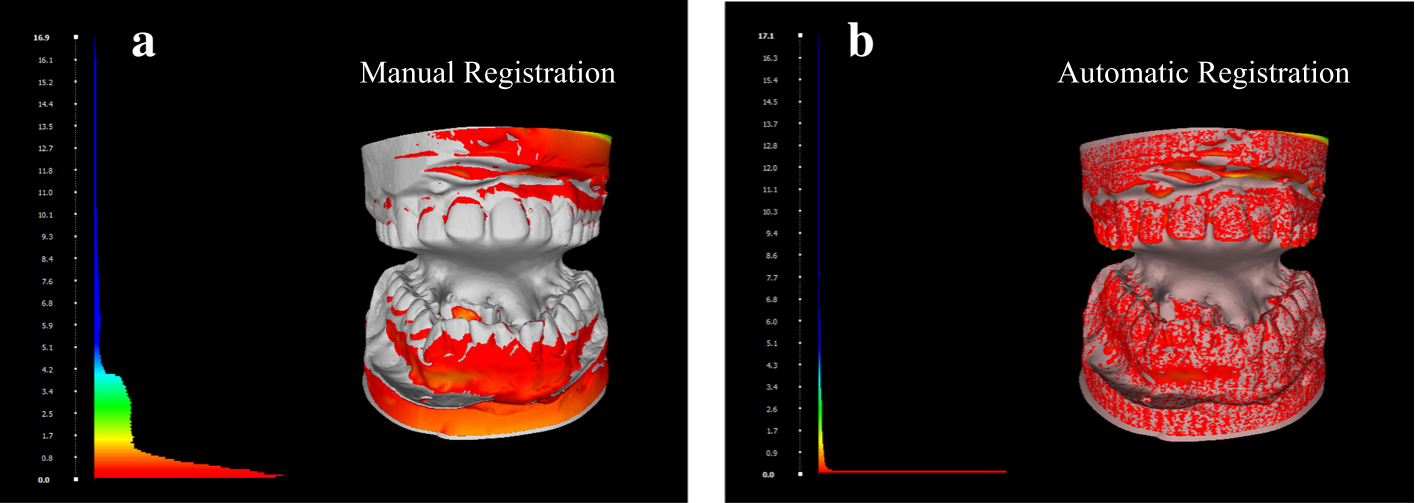
Hausdorff distance computation between the registered meshes and the base mesh, followed by color mapping by vertex quality. **a** manual registration **b** automatic registration using the ICP algorithm

Results showed a statistically significant advantage for the automatic-registration (t-test, *p* < 0.0001) in terms of time and reproducibility (Additional file 1: Figure S3). The mean time taken for manual registration was 15 min per case (range = 10–19, SD = 3.3), and for automatic registration was 3.8 min per case (range = 3–4.5, SD = 0.5). The difference between the aligned meshes was calculated by computing the Hausdorff distance between vertices [16], which projects the vertices from the reference mesh onto the corresponding points on the target mesh and measures the minimum, maximum, and mean distance between vertices. This was followed by color mapping of the vertex quality to achieve a visual representation of the accuracy of fit (Fig. 4). This function utilizes the Red-Green-Blue (RGB) color map, where red means zero error (good registration) and blue means high error (poor registration) [16]. A highly accurate registration will appear as a pattern of red colored vertices on the reference mesh, as depicted in Fig. 4b.

### Fit of the printed stents

**Fig. 5:**
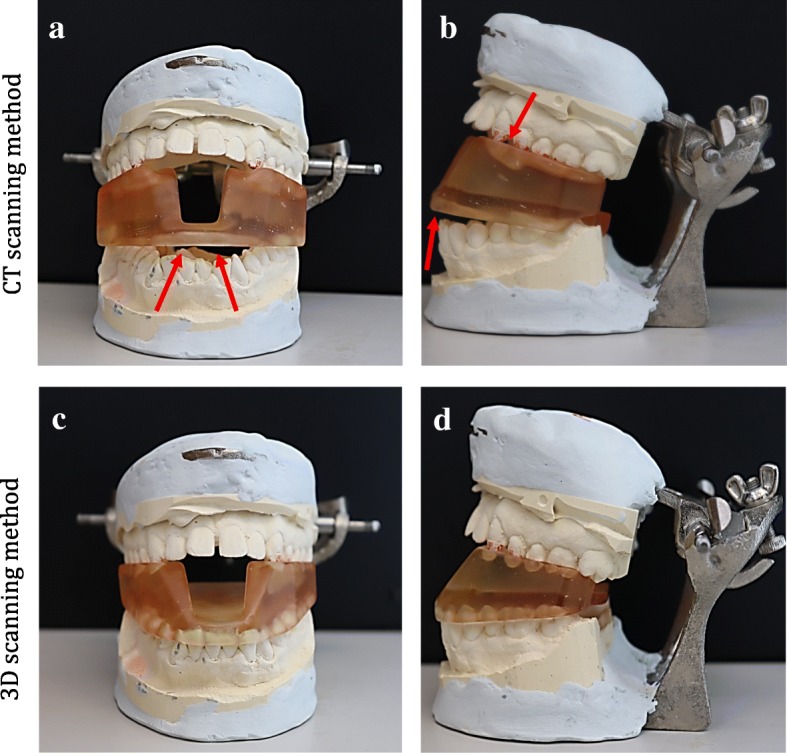
3D-printed stent positioned on the dental stone model of a patient with underbite. **a** & **b** Frontal and lateral view of the stent made from CT scan segmentation. Arrows point to areas where the stent does not fit into the model. **c** & **d** Frontal and lateral view of the stent made using 3D-scanning method

Assessment of the fit of the stents was based on two parameters. First was trueness, which was evaluated by the accuracy of the stent positioning on patients’ teeth, using the stone model. Stents printed using the 3D-scanning workflow successfully accommodated to patients’ teeth, in contrast to stents printed using the CT scanning workflow (Fig. 5). Second was the accuracy in replicating patients’ bites which was evaluated using the articulated stone models with an open vertical dimension of occlusion as the ground truth. The stents fabricated using the 3D-scanning workflow were successful in replicating the patient’s bite to include a case of severe Angle’s Class III malocclusion (case 3). Meanwhile, printed stents using the CT-based workflow failed to accurately replicate the patients’ bite.

## Discussion

In this study, we explored the utility and limitations of 3D-scanning and 3D-printing technologies to design and fabricate custom mouth opening tongue-depressing (MOTD) stents for HNC radiotherapy. The combination of 3D-scanning, CAD, and 3D-printing showed a significant impact on the workflow and efficiency of the design and fabrication processes, which would help broaden their availability. When compared to a previously published CT imaging-based method [13], the 3D-scanning workflow with automatic registration described in this study required significantly less time and efforts. Our new 3D-scanning and printing method has demonstrated a clear advantage in terms of accuracy, reproducibility, and trueness of fit into the stone models.

Radiation stents have been utilized for decades with variable degrees of customization. The conventional customized radiation stents are made by an oral-maxillofacial surgeon from patient's dental impressions, by first making stone models with an appropriate maxillo-mandibular jaw relation record. These models are then used to hand sculpt a wax-pattern stent, which is subsequently used to make the definitive stent made from polymethyl methacrylate (PMMA) resin. This conventional workflow is often challenged by the multiple appointments, intensive labor, time, and experience it dictates. To overcome these limitations, Wilke et al. [13] proposed a novel workflow for radiation stent fabrication through utilizing CAD and 3D-printing from routine diagnostic CT imaging. However, relying on CT imaging to acquire dental anatomy has its limitations. Images are susceptible to various artifacts, notably, metallic and motion artifacts [17–19], and the segmentation process is limited by the time, skill, accuracy, and reproducibility factors. Even with the semi-automated and automated segmentation methods, it is challenging to evaluate the accuracy without a known ground truth, such as impression casts or intraoral scanner (IOS) images. [20, 21]. Hence, CT imaging and segmentation are more prone to inaccurate representations compared to the 3D scanning method. To our knowledge, the workflow described in this study is the first to objectively remedy the addressed concerns. Additionally, it overcomes the challenges associated with anatomical and pathological variabilities such as edentulous patients and those with severe malocclusions. We utilized reliable and validated commercially available equipment and created an efficient workflow widely applicable in practice.

This study exhibits a few limitations. First, the reliance on the stone models to acquire dental anatomy which requires an extra visit to the oral-maxillofacial surgeon. We are investigating the incorporation of IOS to acquire the oral anatomy at the patient’s point of care. IOS has demonstrated superiority over traditional methods of dental impression making in terms of accuracy, dimensional change, retrieval, storage and safety in patients with aspiration risks (cleft-lip and palate) or respiratory distress [22–24]. Another limitation is the reliance on the stone models for outcome evaluation instead of assessing the fit of stents in patients’ mouth and their feedback. Currently, we are conducting a clinical trial to comprehensively assess the efficiency of the proposed workflow in terms of time, labor, and patient-reported-outcomes (PROs). We also plan to apply our digital workflow to other stent designs such as the tongue lateralizing, mouth opening tongue-elevating, and lip protruding stents, which simply require swapping out digital components of the stent design. The tongue lateralizing stent is useful for treating unilateral tonsil cancers, and the mouth opening tongue-elevating stent is useful in treatment of floor of the mouth cancers. The lip protruding stent design is utilized in the treatment of oral cavity cancers such as malignant lesions in the buccal mucosa. We also anticipate that our digital and 3D workflow will lead to economic benefits, and we plan a detailed cost analysis of our method as compared to traditional hand-crafted methods. We acknowledge that lack of complete automation may lead to design variability and delay. Future studies will investigate an automated workflow and personalized design algorithms. Additionally, it is imperative to compare the 3D-printed resin with standard materials like PMMA in mechanical and surface microscopic properties, and radiation sensitivity.

## Conclusions

We have outlined an improved workflow for designing and fabricating a 3D-printed radiation stent for HNC patients using the mouth opening tongue-depressing model. Our results demonstrated the potential advantages of utilizing the 3D-scanning technology to overcome the inherent limitations associated with CT diagnostic imaging. The proposed workflow can be conveniently incorporated into radiation oncology practices, and future studies aiming to evaluate the clinical benefits of customized 3D-printed stents are warranted.

## Additional files

Additional file 1: **Figure S1** A flowchart compares the workflow for the two tested methods; CT images segmentation versus 3D scanning. **Figure S2** Unpaired t-test shows a significant advantage for the 3D scanning method compared to CT derived segmentation in terms of reproducibility measured by the average dice coefficient for the 3 observers. **Figure S3** Unpaired t-test shows a significant advantage for the ICP registration method over the manual one in terms of time efficiency. (PPTX 54 kb)

Additional file 2: **Table S1** compares the dice coefficient between the manually segmented dental structures versus 3D scanned one. (DOCX 15 kb)

## Data Availability

The datasets used and analyzed during the current study are available from the corresponding author upon request.

## Abbreviations

CAD: Computer-aided designing
CT: Computed tomography
DSC: Dice similarity coefficient
HNC: Head and Neck Cancer
ICP: Iterative Closest Point
IOS: Intraoral scanners
IPA: Isopropyl alcohol
MOTD: Mouth opening tongue-depressing
PMMA: Polymethyl methacrylate
PRO: Patient reported outcomes
RGB: Red-Green-Blue
RIOM: Radiation induced oral mucositis
RT: Radiation therapy
STL: Stereolithography
UV: Ultraviolet
