# Supplementary figures and images for: Creating customized oral stents for head and neck radiotherapy using 3D scanning and printing
Source: Radiat Oncol. 2019 Aug 19;14:148. doi: 10.1186/s13014-019-1357-2 (PMC6701083; doi:10.1186/s13014-019-1357-2)

## Slide 1
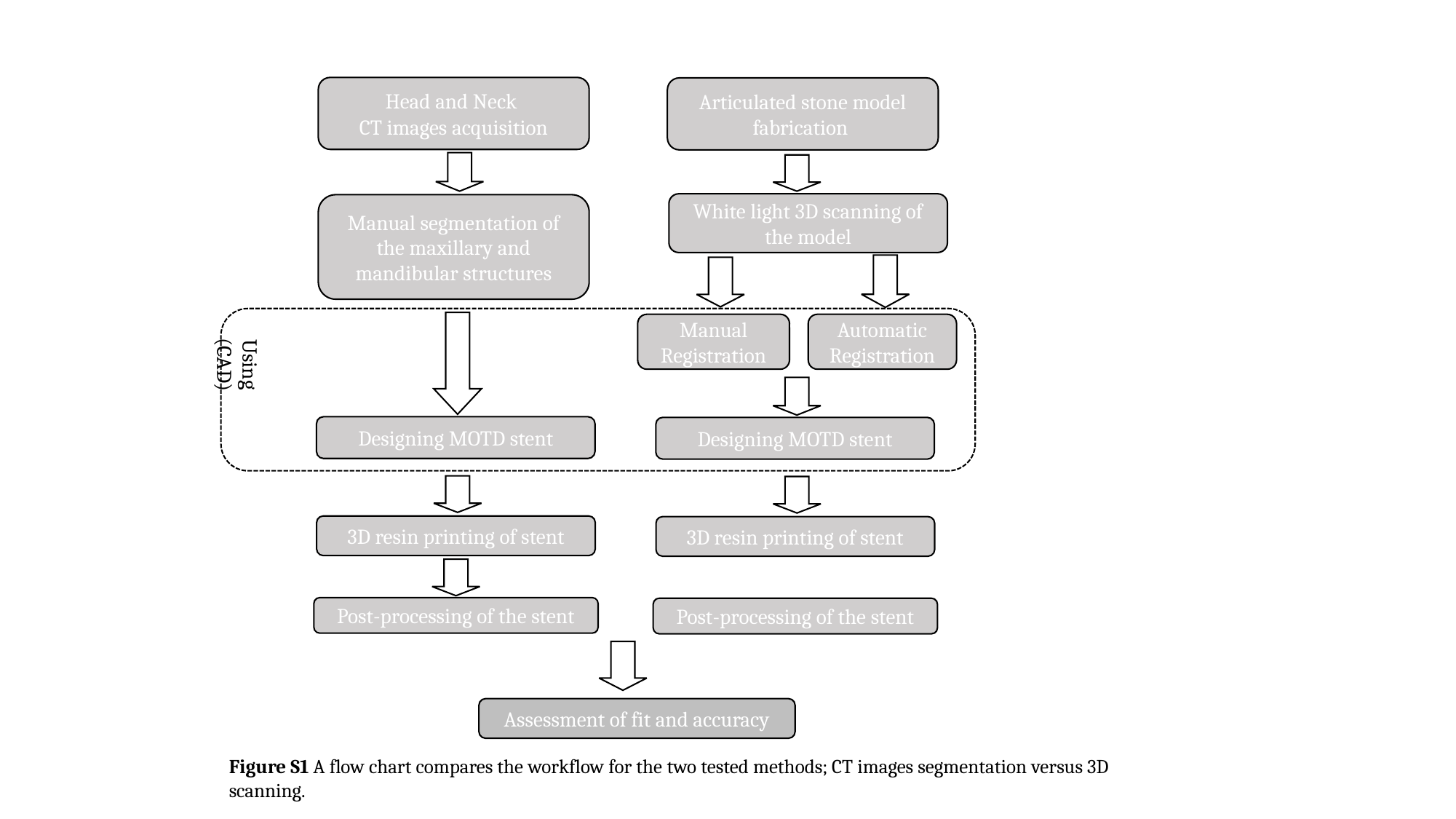

## Slide 2
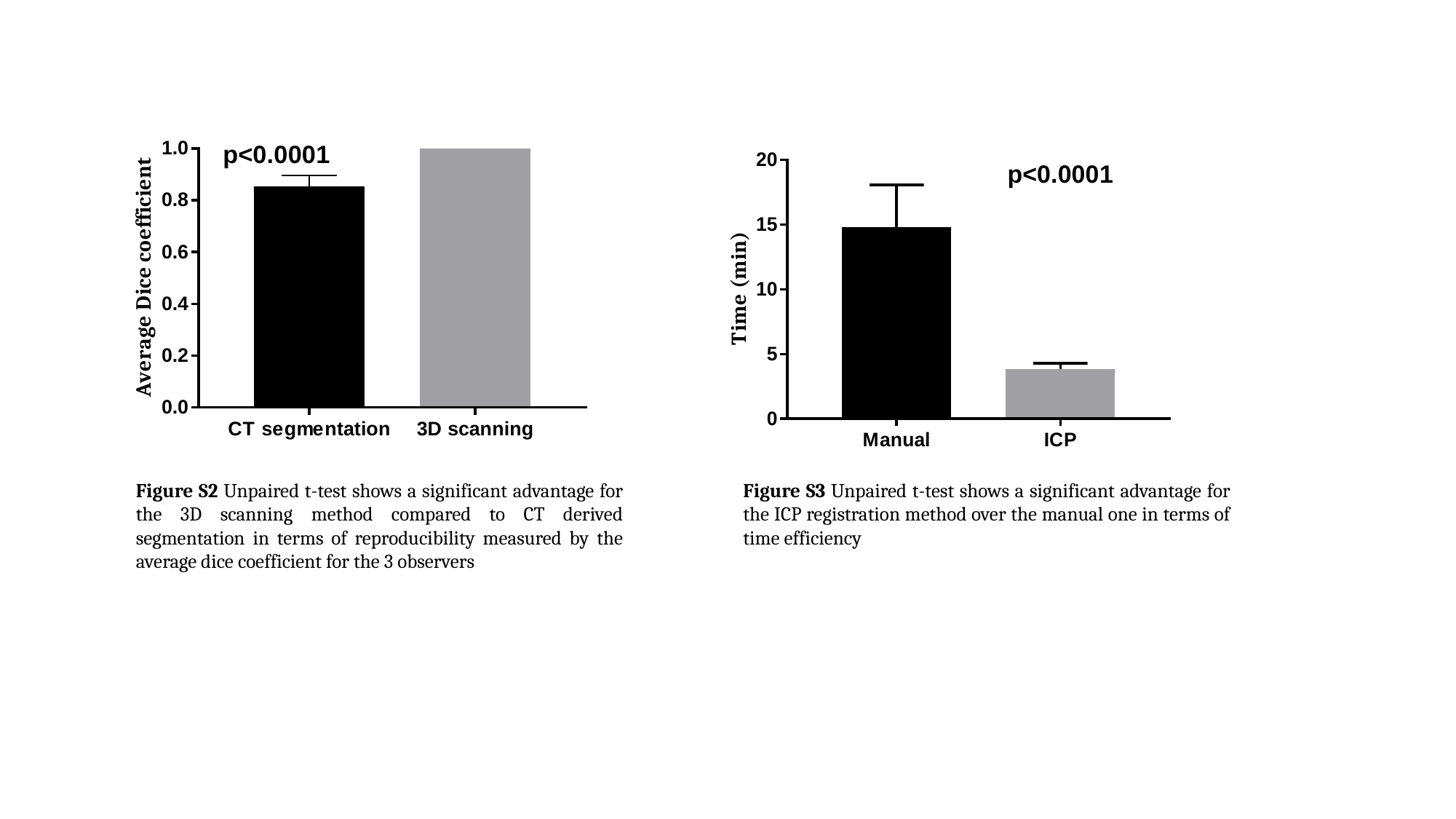

Supplement: Supplementary file 1 — Figure S1 A flowchart compares the workflow for the two tested methods; CT images segmentation versus 3D scanning. Figure S2 Unpaired t-test shows a significant advantage for the 3D scanning method compared to CT derived segmentation in terms of reproducibility measured by the average dice coefficient for the 3 observers. Figure S3 Unpaired t-test shows a significant advantage for the ICP registration method over the manual one in terms of time efficiency. (PPTX 54 kb) [file 13014_2019_1357_MOESM1_ESM.pptx]
