# Supplementary material for: Creating customized oral stents for head and neck radiotherapy using 3D scanning and printing
Source: Radiat Oncol. 2019 Aug 19;14:148. doi: 10.1186/s13014-019-1357-2 (PMC6701083; doi:10.1186/s13014-019-1357-2)
Supplement: Supplementary file 2 — Table S1 compares the dice coefficient between the manually segmented dental structures versus 3D scanned one. (DOCX 15 kb) [file 13014_2019_1357_MOESM2_ESM.docx]

| Table S1 compares the dice coefficient between the manually segmented dental structures versus 3D scanned one | | | | | | | |
| --- | --- | --- | --- | --- | --- | --- | --- |
|  | | **CT Segmentation** | | | **3D scanning** | | |
|  |  | **Observer #1** | **Observer #2** | **Observer #3** | **Observer #1** | **Observer #2** | **Observer #3** |
| **Patient 1** | **Maxilla** | - | 0.885 | 0.843 | - | 1 | 1 |
|  |  | 0.885 | - | 0.878 | 1 | - | 1 |
|  |  | 0.843 | 0.878 | - | 1 | 1 | - |
|  | **Mandible** | - | 0.903 | 0.944 | - | 1 | 1 |
|  |  | 0.903 | - | 0.888 | 1 | - | 1 |
|  |  | 0.944 | 0.888 | - | 1 | 1 | - |
| **Patient 2** | **Maxilla** | - | 0.887 | 0.827 | - | 1 | 1 |
|  |  | 0.887 | - | 0.809 | 1 | - | 1 |
|  |  | 0.827 | 0.809 | - | 1 | 1 | - |
|  | **Mandible** | - | 0.703 | 0.87 | - | 1 | 1 |
|  |  | 0.703 | - | 0.792 | 1 | - | 1 |
|  |  | 0.87 | 0.792 | - | 1 | 1 | - |
| **Patient 3** | **Maxilla** | - | 0.875 | 0.817 | - | 1 | 1 |
|  |  | 0.875 | - | 0.794 | 1 | - | 1 |
|  |  | 0.817 | 0.794 | - | 1 | 1 | - |
|  | **Mandible** | - | 0.87 | 0.915 | - | 1 | 1 |
|  |  | 0.87 | - | 0.847 | 1 | - | 1 |
|  |  | 0.915 | 0.847 | - | 1 | 1 | - |
